# Supplementary material for: A Novel Dipeptidyl Peptidase-4 Inhibitor DA-1229 Ameliorates Tubulointerstitial Fibrosis in Cyclosporine Nephrotoxicity in Mice
Source: Life (Basel). 2021 Mar 18;11(3):251. doi: 10.3390/life11030251 (PMC8003165; doi:10.3390/life11030251)
Supplement: Supplementary file 1 [file life-11-00251-s001.pdf]

life

MDPI

Supplementary Materials

A Novel Dipeptidyl Peptidase-4 Inhibitor DA-1229 Ameliorates Tubulointerstitial Fibrosis in Cyclosporine Nephrotoxicity in Mice

Table S1. Primer sequences for real-time quantitative PCR.

Target Gene	Primer sequence('5-'3)
TLR4, forward	GGGAACAAACAGCCTGAGAC
TLR4, reverse	AGACCCATGAAATTGGCA T
TNF-α, forward	CCGATGGGTTGTACCTTGTC
TNF-α, reverse	GGCAGAGAGGAGGTTGACTTT
IL-1β, forward	CTC ACA AGC AGA GCA CAA GC
IL-1β, reverse	ACG GAT TCC ATG GTG AAG TC
MCP-1, forward	CTGGATCGGAACCAAATGAG
MCP-1, reverse	CGGGTCAACTTCACATTCAA
PAI-1, forward	TCCTCATCCTGCCTAAGTTCTC
PAI-1, reverse	GTGCCGCTCTCGTTTACCTC
TGFβ1, forward	AGCCCGAAGCGGACTACTAT
TGFβ1, reverse	CTGTGTGAGATGTCTTTGGTTTTC
Col-I, forward	CCAAAGGTGCTGATGGTTCT
Col-I, reverse	ACCAGCTTCACCCTTGTCAC
CTGF, forward	TGGCGAGATCATGAAAAAGA
CTGF, reverse	AGATGTCATTGTCCCCAGGA
Col-IV, forward	GCTCTGGCTGTGGAAAATGT
Col-IV, reverse	CTTGCATCCCGGGAAATC
HMGB1, forward	AACCTGATGCAGCGAAAAAG
HMGB1, reverse	CCTCATCCTCTTCATCCTCCT
β -actin, forward	GGACTCCTATGTGGGTGACG
β -actin, reverse	CTTCTCCATGTCGTCCCAGT

TLR4, Toll likre receptor 4; TNF-α , Tumor necrosis factor-α; IL-1, interleukin-1; MCP-1, monocyte chemoattractant peptide-1; PAI-1, plasminogen activator inhibitor-1; TGFβ, transforming growth factor; Col-I, type I collagen; CTGF, connective tissue growth factor; Col-IV, type IV collagen; HMGB1, high-mobility group protein B1. In this experiment, each sample was run in triplicate, and the corresponding non-reverse transcribed mRNA samples were used as negative controls. The mRNA level of each sample was normalized to that of β-actin mRNA.
